# Editorial: Viral infection pathogenesis and pathology in nervous system

**DOI:** 10.3389/fcimb.2025.1554095

**Published:** 2025-02-12

**Authors:** A. Alwin Prem Anand

**Affiliations:** Independent Researcher, Madurai, Tamil Nadu, India

**Keywords:** chikungunya virus, herpes simplex virus, IRF1, blood brain barrier, extracellular vesicles (EVs), Borna disease virus, mental health

Viral infections have profound impact on human health, ranging from acute systemic effects to chronic neurological complications. The interplay between viruses and the host immune system is pivotal in determining disease outcomes. Several viruses that infect the nervous system have been clinically and experimentally proven. To under the pathogenesis and pathology of viral infections in nervous system, it is essential to understand their molecular mechanisms, immunopathology, immune evasion strategies, methods of crossing the blood-brain barrier (BBB) and the roles of non-coding RNAs (miRNA, lncRNA, etc) in disease process. The current Research Topic “*Viral infection pathogenesis and pathology in nervous system*” and its collection of five article provides insights into abovementioned aspects.

## Molecular mechanisms in neuropathogenesis and neuropathology

Understanding molecular mechanisms is essential for elucidating the role of viruses in neuropathology and neuropathogenesis. Kumar et al. and Liu et al., have experimentally demonstrated mechanistic insights into infections caused by Chikungunya virus (CHIKV) and Herpes simplex virus (HSV)-1, respectively.

In CHIKV, neurological complications such as encephalitis, Guillain-Barré syndrome and meningoencephalitis have been reported (Freppel et al., 2024; Mehta et al., 2018). Experimental CHIKV infection have shown perturbed glia-neuron interaction (Inglis et al., 2016). Astrocytes (Abraham et al., 2013) and microglia cells (Abere et al., 2012; Qadri et al., 2022; Wintachai et al., 2012) have been used to investigate the neuropathogenesis of CHIKV. Kumar et al. studied CHIKV infection in microglial cells, providing evidence that CHIKV induces apoptosis, altered surface markers such as CD11c, CD14, and HLA-DR, and causes mitochondrial membrane depolarization. They also observed the presence of immature virions at 24 hpi (hours post infection) within cytoplasmic vesicles and adjacent to the inner and outer surfaces of the cell membrane. Additionally, cluster of mature virions were observed being released from infected cells at 48 hpi.

Liu et al. experimentally showed that IRF1 is a critical regulator of HSV-1 replication in human astrocytes. IRF1 knockout (KO) cell lines result in increased expression of HSV-1 genes. Additionally, it was observed that IRF1 overexpression can enhance poly(dA:dT)-driven inhibition of HSV-1 replication. Taken together, this suggests that HSV-1 suppress IRF1 expression. The role of IRF1 in relation to HSV-1 infection was least studied with very few articles (Ru et al., 2014; Wang et al., 2019; Xie et al., 2018) addressing IRF1 and HSV-1. Similar to Liu et al., IRF1 KO keratinocyte cell lines also show increased expression of HSV-1 genes (Wang et al., 2019). Experimental evidence indicates that reduced IRF1 expression enhances the HSV-1 replication, and this reduction is due to miRNA regulation of IRF1 gene, particularly miR-23a and miR-373 (Ru et al., 2014; Xie et al., 2018). Interestingly, IRF1 is necessary for the induction of innate immune response in humans, as opposed to mice (Wang et al., 2019). Taken together, these findings suggest that HSV-1 evades immune activation by suppressing IRF1, warranting further investigation.

## Immune evasion mechanism in Alphaviruses

The Alphaviruses such as CHIKV, Western equine encephalitis virus (WEEV), Venezuelan equine encephalitis virus (VEEV), and Eastern equine encephalitis virus (EEEV) cause neurological complications. Atypical manifestations of CHIKV with neurological complications are often fatal (Economopoulou et al., 2009; Tandale et al., 2009) and can cause Guillain-Barré syndrome (Lebrun et al., 2009). Recent experimental evidence shows CHIKV disrupts the integrity of the BBB and crosses into the brain (de Souza et al., 2024). The review by de Oliveira Souza et al. have detailed on how alphaviruses (CHIKV, WEEV, VEEV, EEV) evade immune surveillance and cause neuropathogenicity. Fascinatingly, the alphaviruses WEEV, VEEV and EEEV can invade brain in regions lacking BBB, such as the olfactory bulb and circumventricular organs.

## Extracellular vesicles in viral neuropathogenesis

Currently, extracellular vesicles (EVs) are captivating topic among researchers due to their potential use as diagnostic tools in cancer (Kumar et al., 2024), neurological and neurodegenerative disorders (Li et al., 2023; Zanirati et al., 2024), and viral infections (McNamara and Dittmer, 2020). EVs have been reported in several neurotrophic viruses, including Zika virus (ZIKV) (Huang et al., 2018), Japanese encephalitis virus (JEV) (Mukherjee et al., 2019), HSV-1 (Sun et al., 2024), JC polyomavirus (JCPyV) (Morris-Love et al., 2019, Morris-Love et al., 2022; O’Hara et al., 2020; Oberholster et al., 2023) and HIV (Hu et al., 2020). Oberholster et al. reviewed the possibility of using brain-derived EVs to develop biomarkers during viral infections. From their review, it is evident that viruses can utilize EVs to 1) act as encapsulation to evade immune detection (HSV-1, ZIKV), 2) enhance the efficiency of infection (ZIKV), 3) serve as a trojan horse by delivering viral components into neighbouring cells (JEV, JCPyV, HSV-1), 4) modulate host proteins to make cells carry viral proteins (eg. Nef protein from HIV), 5) to compromise integrity and permeability of BBB (ZIKV, JEV, HSV-1, and JCPyV), 6) deliver components that cause neuronal injury or inflammation (e.g., JEV - EVs activate caspases, leading to neuronal damage, and HIV - EVs containing viral proteins induce oxidative stress and synaptic injury).

## Viral infections, CNS pathology, and mental health

Several viruses cause CNS infection which may result in encephalitis; however, its effects on mental disorders or neuropsychiatric illnesses are among the least studied. Neurotrophic viruses found in brain tissue and cerebrospinal fluid include *influenza A virus* (Frankova et al., 1977; Ishigami et al., 2004), HSV (Genet et al., 2023; Nicoll et al., 1991), CMV (Yagmur et al., 2016; Zheng et al., 2023), rubella virus (RV) (Lazar et al., 2016; Monif et al., 1965), Epstein-Barr virus (EBV) (Hassani et al., 2018; Huang et al., 2021; Kobayashi et al., 2008), HIV (Wahl and Al-Harthi, 2023; Williams and Naude, 2024), SARS-CoV-2 (Gomes et al., 2021; Stein et al., 2022) and Borna disease virus (BoDV) (Bode et al., 1996; de la Torre et al., 1996; Eisermann et al., 2021; Nakamura, 1998; Nakamura et al., 2000; Niller et al., 2020). Lorkiewicz and Waszkiewicz, in their review, have explored the cytokine profiles of these viruses and compared it with mental/psychiatric disorders including major depressive disorder, bipolar disorder (BD), schizophrenia (SCZ), obsessive compulsive disorder, posttraumatic stress disorder, autism spectrum disorder, suicide and first episode psychosis. According to their conclusion, 1) prenatal exposure to influenza and HSV-2 elevates the risk for SCZ. 2) CMV and EBV exhibit potential links to depression and BD, though EBV’s association remains inconclusive and requires further investigation. 3) BoDV uniquely aligns with affective disorders, reflecting its symptomatology in animals, while 4) HIV has a well-documented prevalence of cognitive and mental disorders among patients. 5) RV suggests possible mental health implications based on its neurological mechanisms. 6) SARS-CoV-2 infection is associated with an increased, albeit time-limited, risk of mental illnesses, highlighting its acute rather than chronic impact. In the case of cytokine expression profiles, it is impossible to link them to any mental disorder, due to the broad and nonspecific nature of cytokine expression during viral infection. Obtaining infectious viruses from postmortem brain tissue and detecting viral gene expression within the brain suggest that these viruses may play a role in modulating pathways that could lead to psychiatric illnesses or exacerbate the severity of existing ones, warranting further study.
